# Spatial Modeling of the Potential Distribution of Dengue in the City of Manta, Ecuador

**DOI:** 10.3390/ijerph22101521

**Published:** 2025-10-04

**Authors:** Karina Lalangui-Vivanco, Emmanuelle Quentin, Marco Sánchez-Murillo, Max Cotera-Mantilla, Luis Loor, Milton Espinoza, Johanna Mabel Sánchez-Rodríguez, Mauricio Espinel, Patricio Ponce, Varsovia Cevallos

**Affiliations:** 1 Centro de Investigación en Salud Pública y Epidemiología Clínica (CISPEC), Facultad Ciencias de la Salud Eugenio Espejo, Universidad UTE, Quito 170527, Ecuador; 2 Instituto Nacional de Investigación en Salud Pública, INSPI Dr. Leopoldo Izquieta Pérez, Centro de Investigación EpiSIG, Quito 170136, Ecuador; ma_sm@hotmail.com (M.S.-M.); max_vgom@hotmail.com (M.C.-M.); 3 Facultad Ciencias de la Salud, Carrera de Medicina, Universidad Laica Eloy Alfaro de Manabí, Manta 130222, Ecuador; lcdoluis_84@hotmail.com (L.L.); milton.espinoza@uleam.edu.ec (M.E.); dramabelsan24@hotmail.com (J.M.S.-R.); espinel.mauricio@yahoo.com (M.E.); 4 Facultad Ciencias de la Salud, Carrera de enfermería, Universidad Estatal del Sur de Manabí, Manta 130104, Ecuador; 5 Instituto Nacional de Investigación en Salud Pública, INSPI Dr. Leopoldo Izquieta Pérez, Centro de Investigación CIREV, Quito 170136, Ecuador; wpponcey@yahoo.com (P.P.); vcevallos@inspi.gob.ec (V.C.)

**Keywords:** arboviral infectious diseases, spatial modeling, dengue, Ecuador

## Abstract

In Ecuador, the transmission of dengue has steadily increased in recent decades, particularly in coastal cities like Manta, where the conditions are favorable for the proliferation of the Aedes aegypti mosquito. The objective of this study was to model the spatial distribution of dengue transmission risk in Manta, a coastal city in Ecuador with consistently high incidence rates. A total of 148 georeferenced dengue cases from 2018 to 2021 were collected, and environmental and socioeconomic variables were incorporated into a maximum entropy model (MaxEnt). Additionally, climate and social zoning were performed using a multi-criteria model in TerrSet. The MaxEnt model demonstrated excellent predictive ability (training AUC = 0.916; test AUC = 0.876) and identified population density, sewer system access, and distance to rivers as the primary predictors. Three high-risk clusters were identified in the southern, northwestern, and northeastern parts of the city, while the coastal strip showed lower suitability due to low rainfall and vegetation. These findings reveal the strong spatial heterogeneity of dengue risk at the neighborhood level and provide operational information for targeted interventions. This approach can support more efficient surveillance, resource allocation, and community action in coastal urban areas affected by vector-borne diseases.

## 1. Introduction

Dengue fever, transmitted primarily by the mosquito *Aedes aegypti*, remains one of the most important vector-borne diseases worldwide, with an estimated 400 million infections per year and an expanding geographic footprint driven by urbanization and climate change [1]. Over the past decades, Ecuador has experienced a gradual but sustained expansion of endemic dengue transmission, with the coastal cities bearing the heaviest burden, driven in large part by the urbanization and connectivity of its coastal hubs. Katzelnick et al. [2] showed that coastal provinces with major cities—including Manabí, home to Manta—were among the first to reach high force-of-infection levels around 1980 and have maintained elevated incidence ever since. By applying a species-distribution framework to over 11,000 *Aedes aegypti* presence records, they identified population size, waste removal, elevation and water access as the strongest predictors of vector habitat suitability for Ecuador [2]. The province of Manabí has been categorized as a high-risk area for Aedes aegypti. Dengue cases from this province accounted for 28.6% of the national total in 2018, 15.3% in 2019, 18.7% in 2020, and 15.5% in 2021 [3].

At a household-level scale, Talbot et al. [4] conducted entomological and socio-environmental surveys in Manta, Ecuador, alongside Ibagué, Colombia, and Posadas, Argentina, to identify key determinants of arbovirus transmission risk across diverse urban contexts and found that factors such as greater household occupancy, structural entry points for mosquitoes and ornamental vegetation significantly increased *Ae. aegypti* female density, whereas higher socioeconomic status, arbovirus awareness and routine container management practices reduced vector abundance. These findings underscore that intra-urban heterogeneity in housing conditions and water management practices creates local “hotspots” of transmission risk.

Manta, a major commercial port, routinely records dengue incidence rates above the national average, particularly during the January–June rainy season when vector breeding intensifies. Addressing dengue in Manta therefore requires interventions that operate at both the macro-environmental scale—responding to broad trends in urbanization and infrastructure—and the micro-ecological scale—specific social and environmental determinants of *Aedes aegypti* abundance in the city.

In this work, we address this gap by modeling the spatial distribution of dengue suitability in Manta, with the aim of identifying priority intervention zones and informing targeted vector-control and public health planning. Our integrated approach combines environmental and socio-demographic variables to map habitat suitability for Aedes aegypti and translates these insights into actionable recommendations. By bridging large-scale transmission modeling with locally relevant indicators, this study offers a practical framework to support surveillance and control strategies in coastal urban settings.

## 2. Materials and Methods

### 2.1. Study Area

**Figure 1:**
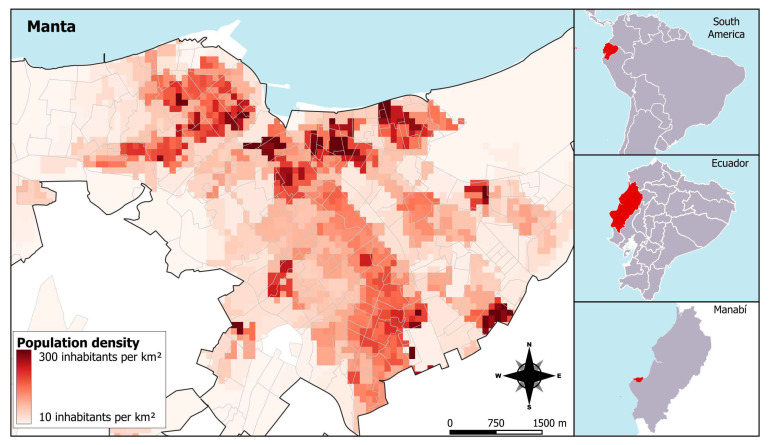
Map of the study area and population density in Manta, 2020. The color scale represents population density (inhabitants per km^2^), where darker shades indicate higher concentrations. Population data were obtained from the population projections of the Instituto Nacional de Estadística y Censos (INEC).

The city of Manta is located on Ecuador’s central coast (Figure 1), with an approximate population of 261,871 inhabitants [5]. It is one of the main urban centers in the province of Manabí and a significant maritime hub in the country. Manta has a subtropical to maritime temperate climate, with average annual temperatures ranging between 21 °C and 28 °C, and a climatic pattern characterized by a rainy season from January to June and a dry season from July to December. The city features diverse infrastructure, including high-density urban sectors and marginalized neighborhoods with limited access to basic services.

### 2.2. Data Sources

The species distribution model used dengue cases from 2018 to 2021, which were obtained from the Epidemiological Surveillance System (ViEpi) database of the Ministry of Public Health (MSP). Under a confidentiality agreement, the residential address of each case was obtained, enabling approximate georeferencing. Only the most precisely georeferenced cases were included, resulting in a total of 148 (54%) georeferenced dengue cases. While this represents a subset of the total number of cases, the number of georeferenced records is consistent with recommendations in the ecological niche modeling literature, where MaxEnt has demonstrated robust performance even with much smaller sample sizes (10–30 records), and predictive accuracy becomes more stable when more than 100 records are available. [6,7]. The initial database contained no personal or individual information that could identify any case.

A geographic database was compiled using several freely available sources to construct the explanatory variables. In particular, population and housing census data, located by census tract, came from the Instituto Nacional de Estadística y Censos (INEC) and was publicly available via the institution’s website, free of any personal identifiers. Satellite images for environmental layers were mainly downloaded from the open-access platform https://search.earthdata.nasa.gov/ (accessed on 27 June 2025). Table 1 provides a detailed list of all variables used. All layers were processed at a spatial resolution of 1 hectare (100 m × 100 m).

Before constructing the model, the monthly environmental variables were synthesized into a single principal component using Principal Component Analysis (PCA). This approach was employed to reduce the high correlation among variables, which is not recommended in species distribution models [8]. The first principal component was selected as it captures the variability most closely related to the ecology of the disease vector.

### 2.3. Spatial Modeling of the Potential Distribution of Dengue

Using georeferenced data and explanatory variables influencing the distribution of the dengue vector, the maximum entropy model (MaxEnt) was applied to generate probabilistic maps identifying regions with the most favorable conditions for the presence of the dengue infectious agent. MaxEnt 3.3.3 was chosen due to its widespread use and demonstrated effectiveness as one of the best models for species distribution [9].

The model was implemented through a Geographic Information System (GIS), specifically using TerrSet 18.31 [7,10], which includes advanced time-series operations and various options for species distribution modeling. Control parameters were set as follows: 75% of presence points used for training, 25% for validation, 10 iterations (bootstrapping), and a threshold rule of 10 percentile training presence. The receiver operating characteristic (ROC) curve method was applied to evaluate the overall predictive performance of the model. This metric assesses the model’s ability to differentiate between presence and background, where a value of 1 represents a perfect prediction, while a value of 0.5 indicates a prediction no better than random chance [11].

Based on the probability map generated by MaxEnt, a favorable zones map was derived using the zonal statistics tool in QGIS 3.34.7. The mean probability values of all pixels within the boundaries of each neighborhood were calculated, and these averages were subsequently categorized into three suitability classes: high, medium, and low.

In this study, the term ‘suitability’ refers to the output of the Maxent model, which estimates the environmental favorability for the occurrence of dengue cases. Although ‘suitability’ does not directly measure transmission risk, it is used here as a proxy, under the assumption that higher environmental suitability is associated with increased dengue transmission risk.

### 2.4. Multicriteria Model for Zoning Climatic and Social Variables

To contextualize the results of the habitat suitability model, climatic and social zoning was carried out in the city of Manta. The climatic zoning was based on variables such as precipitation and vegetation index, while the social zoning considered socio-economic factors such as access to sewer system, drinking water and housing conditions. The TerrSet Spatial Decision Modeling (SDM) module was used. The model is based on a fuzzy operation to standardize the variables on a suitability scale from 0 to 1 (0 being optimal, 1 being worst). Finally, to generate the final zoning, the mean value of each census tract was extracted, and this continuous image was reclassified into three levels: high, medium and low.

## 3. Results

### 3.1. Potential Habitat Distribution

**Figure 2:**
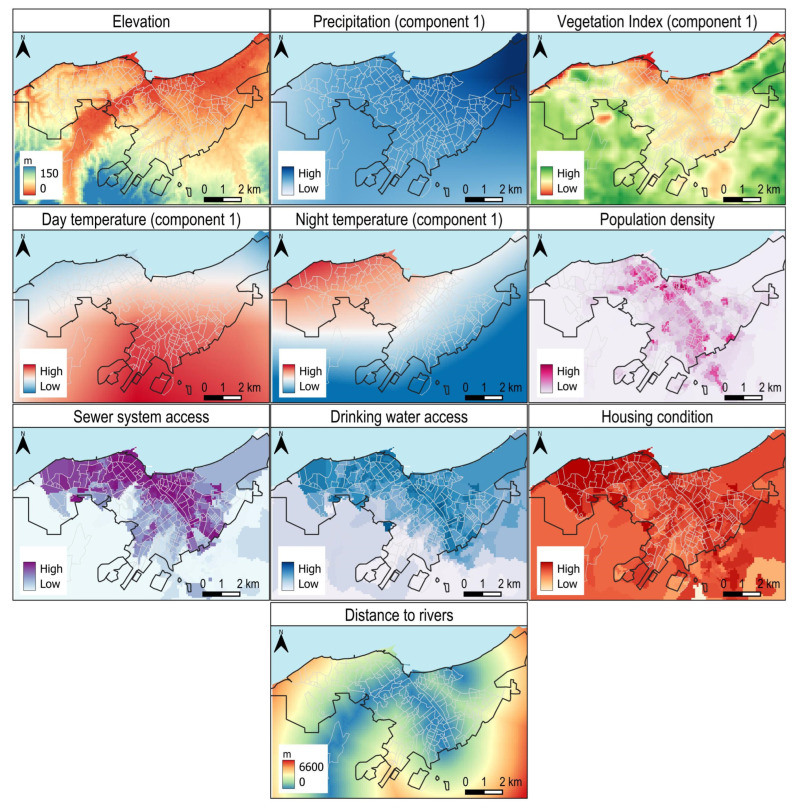
Variables used in the Maxent model to estimate suitability for dengue transmission. These include elevation, first principal component of precipitation, first principal component of vegetation index, first principal component of day temperature, first principal component of night temperature, population density, access to sewer system, access to drinking water, housing condition, and distance to rivers. These variables were selected as potential environmental and socio-economic drivers influencing the spatial distribution of dengue risk. The input values of all variables summarized by neighborhood are provided in Supplementary Materials (File S1).

We used ten variables in the model (Figure 2), and the area under the ROC curve (AUC) for dengue showed an average training value of 0.916 and a test AUC of 0.876 for replicated runs.

**Figure 3:**
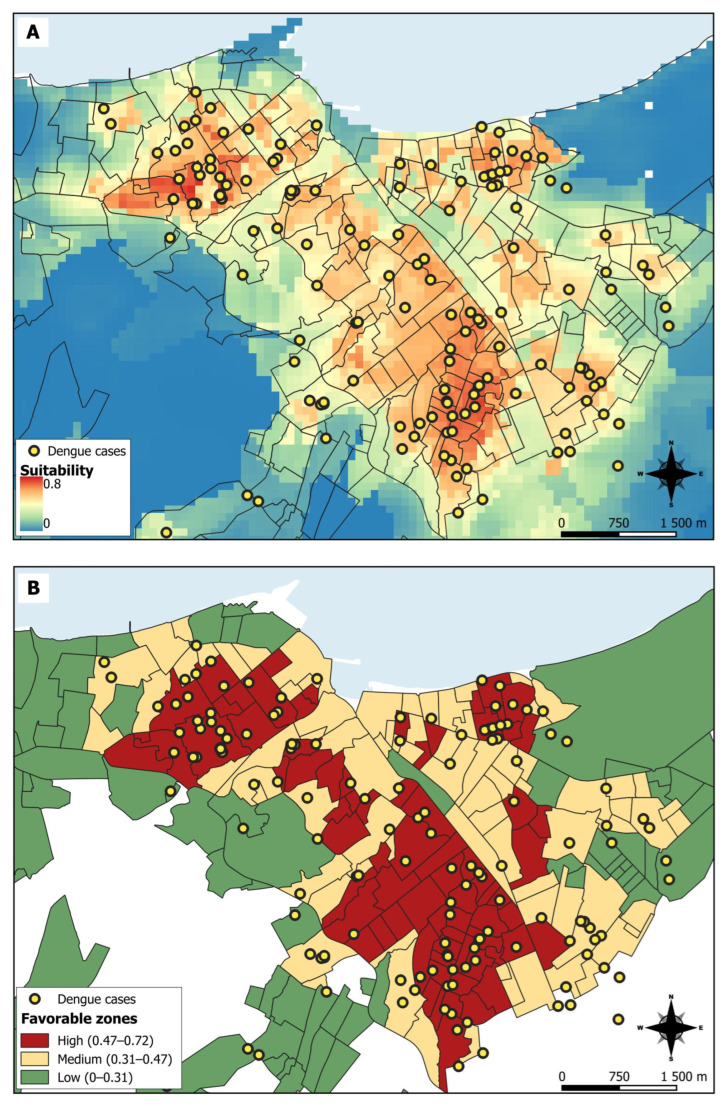
Results from the Maxent habitat suitability model for dengue transmission in Manta. Map (**A**) displays the continuous suitability values, where higher values indicate higher suitability for dengue occurrence (increased risk of infection). Map (**B**) shows the reclassification of Map (**A**) into three categorical risk zones (high, medium, and low) for dengue transmission.

The model output is a continuous probability map representing suitable areas for dengue cases presence. Red indicates a high probability of favorable conditions for the species, orange to yellow indicates areas with typical conditions where the species is found, and lighter shades of green to blue indicate a low probability of meeting the most suitable conditions (Figure 3A).

According to the risk thresholds (favorable zones), most neighborhoods in Manta fall within the high to medium risk levels, while peripheral areas of the city show low risk. The highest probability of disease presence was in neighborhoods in the south and northwest of Manta, and some neighborhoods in the northeast (Figure 3B).

Table 2 presents metrics for high, medium, and low suitability zones. The high suitability areas encompass 17.87% of the study area, yet account for 47.21% of the population and 58.3% of all dengue cases, exhibiting the highest case density (0.088 cases/ha). Areas exhibiting medium suitability comprise 22.24% of the total area, 38.58% of the total population, and 29.17% of the total cases (0.036 cases/ha). Conversely, regions exhibiting low suitability account for 59.90% of the territory, yet comprise only 14.21% of the population and 12.5% of cases, exhibiting a significantly lower density of 0.006 cases per hectare.

**Figure 4:**
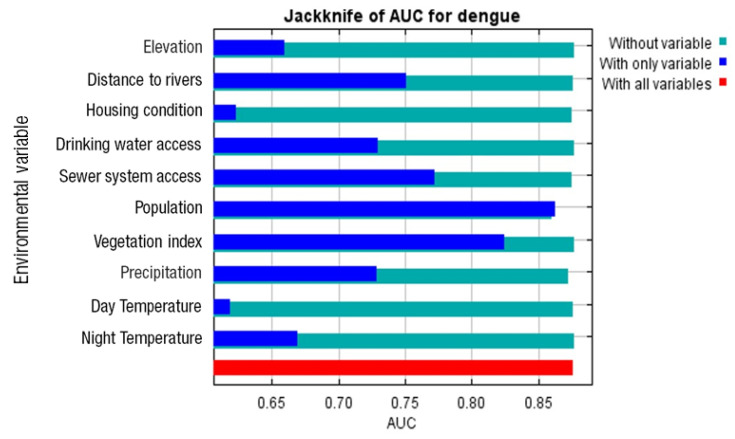
Jackknife test results of regularized gain for the respective explanatory variables used in the Maxent habitat model for dengue transmission.

The model also shows the relative importance of each variable based on the Jackknife test results. The vertical axis represents the explanatory variables, and the horizontal axis represents their scores. The dark blue column indicates the model score when only the variable in question is used, the light blue column represents the sum of scores from other variables, excluding the variable in question, and the red column represents the sum of scores from all variables. Population density was identified as the variable with the highest gain when used in isolation, suggesting it contains the most unique information not present in other variables (Figure 4).

The MaxEnt model also revealed heterogeneous contributions among the ten explanatory variables (see Table 3). The largest contributing factor was population density (72.3%), followed by access to sewerage (13%). Other factors, including distance to rivers (2.5%), nighttime temperature (2.4%), and access to drinking water (2.4%), had more modest contributions. The remaining variables (precipitation, housing condition, vegetation index, altitude, and daytime temperature) each contributed less than 2% of the model’s total gain.

### 3.2. Climatic and Social Zoning

A multi-criteria evaluation process was used to combine the variables, as implemented in the TerrSet system. The first step consisted in re-scaling over the range [0–1] all the variables, parameterizing the fuzzy operation with the linear function so that lower values would be more conducive to disease transmission and high values concern less vulnerable areas.

**Figure 5:**
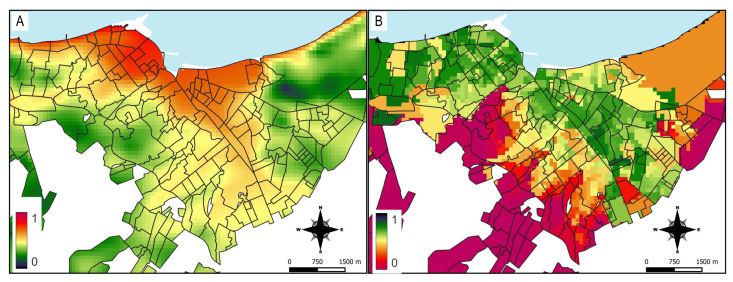
Climatic and social vulnerability zoning in Manta. Map (**A**) represents the climatic vulnerability index: lower values (green) indicate more favorable conditions for dengue transmission, while higher values (red) correspond to less favorable climatic conditions. Map (**B**) represents the social vulnerability index: lower values (red) denote populations with higher social vulnerability factors whereas higher values (green) reflect better social conditions reducing vulnerability to dengue infection.

The climatic zoning shows that the index is high in the coastal area (less rainfall and vegetation), indicating that it would be less favorable for the mosquito and therefore for dengue transmission (Figure 5A). The social zoning in the city of Manta seems to reinforce the climatic zoning, since the areas with better social conditions (higher values) are in the coastal strip and in the urban area of the city. Conversely, areas with higher social vulnerability (lower values) are concentrated in the south and south-east or in peri-urban areas (Figure 5B).

## 4. Discussion

Over recent decades, the global burden of mosquito-borne arboviruses—including dengue, Zika, chikungunya, and yellow fever—has increased sharply in both prevalence and clinical severity. This acceleration, fueled by urbanization, climate variability, and intensifying human mobility, underscores the urgent need to refine and locally tailor emerging Aedes-control technologies so that public health programs can keep pace with an increasingly complex transmission landscape.

Our study set out to test the hypothesis that dengue risk in Manta is spatially heterogeneous and that high-resolution mapping would expose neighborhood-scale hotspots suitable for priority action. The findings confirm this premise and resonate with a growing body of urban dengue-modeling literature.

### 4.1. Hotspots and Their Epidemiological Significance

Three discrete clusters of high suitability—situated in the south, north–west, and north–east of the city—stood out on the probability surface. Similar intra-urban aggregations have been described in Toluca (Mexico) [12] and Malang (Indonesia) [13], where high-risk wards are embedded within wider urban matrices of moderate or low suitability. Collectively, these patterns indicate that fine-scale socio-environmental mosaics, rather than city-wide averages, determine where transmission is most likely to intensify.

### 4.2. Population Density as the Dominant Driver

Jackknife analysis identified population density as the single variable with the greatest unique contribution to model gain, echoing results from the southeastern United States and the Central Mexican Highlands, where incorporating human-density metrics substantially boosted predictive performance over climate-only baselines [14]. High-density blocks increase the host-to-vector ratio, shorten flight distances between mosquitoes and humans, and are often associated with container proliferation—factors that synergistically amplify transmission. These insights call for urban-planning measures (e.g., regulated water storage, solid-waste management, housing improvements) to be embedded within vector-control portfolios.

### 4.3. Coastal Gradient and Climatic Buffering

Climatic zonation revealed lower suitability along the immediate coastline, where reduced rainfall and sparse vegetation constrain larval habitats (see Component 1 of Precipitation and of Vegetation Index in Figure 2). Comparable coastal ‘buffers’ have been documented in other Pacific and Atlantic port cities, where sea-breeze-driven desiccation can impair *Aedes aegypti* survival [15]. Nevertheless, several inland high-risk barrios also suffer from poor water supply and sewer system infrastructure, underscoring that climatic favorability alone does not dictate risk; infrastructural deficits modulate and often magnify it.

### 4.4. Operational Implications for Dengue Control in Manta

The spatial analysis of dengue transmission suitability in Manta reveals several key operational considerations for improving the effectiveness and efficiency of vector control efforts:

Stratified response. The hotspot map supports a tiered intervention plan: intensified larval-source management, targeted ULV spraying, and continuous entomological monitoring in high-suitability clusters. Control decisions in high-risk zones should rely on up-to-date information on insecticide resistance and active vector surveillance. Interventions should be carried out weekly to reduce vector populations. Allocation of resources can be prioritized proportionally to these areas for effective active surveillance and control measures [16]. Additionally, community engagement should be implemented to address the destruction of mosquito breeding containers and improve water storage management.

In medium-risk zones, biweekly surveillance may be suitable to maximize resources for effective control measures and establish community involvement to eliminate breeding sites and water management.

In low-risk peripheral areas, surveillance activities may be conducted monthly, supported by community participation to prevent vector proliferation.

Resource optimization. Focusing efforts where suitability exceeds 70% could markedly reduce operational costs and personnel time relative to blanket city-wide campaigns.

Health-system preparedness. Clinics serving hotspot catchments should bolster diagnostic capacity and stockpile rapid tests ahead of the rainy season to anticipate incidence spikes.

Current Initiatives of the Ministry of Public Health. In Ecuador, dengue control is coordinated by the MSP through integrated strategies of epidemiological surveillance, vector control, and community participation. The MSP has reported investments in insecticides and vector control equipment, distribution of insecticide-impregnated bed nets, and training of over 23,000 health professionals in dengue management [17]. While these initiatives represent important progress, challenges remain in cities such as Manta, where entomological surveillance is still limited and resources are often allocated uniformly rather than according to neighborhood-level risk. Integrating the maps generated in this study with existing MSP strategies could improve targeting, resource efficiency, and overall impact in high-incidence neighborhoods.

### 4.5. Limitations

This study has several limitations that should be considered when interpreting the results. Firstly, the analysis was based on 148 highly accurate georeferenced dengue cases, representing around half of all cases reported in Manta between 2018 and 2021. While this exceeds the recommended threshold for MaxEnt models, the sample size may not accurately reflect all reported cases, particularly in areas with incomplete addresses or missing records.

Secondly, using 2010 socioeconomic data (e.g., access to sewer system and water) to analyze cases from 2018 to 2021 introduces a temporal mismatch that could affect the accuracy of our findings. Although this was the only available dataset at the time of the study, significant changes may have occurred in the intervening years. For instance, infrastructure improvements in Manta, such as the installation of new sewer systems, may have reduced the risk of disease in previously marginalized areas. In the absence of more recent data, however, we assumed that the relative differences between neighborhoods remained consistent over time. This assumption enables comparative analysis across the city, even if the absolute values may have changed. Nevertheless, updated socioeconomic data would enhance the robustness of future studies and enable more precise evaluations of the relationship between infrastructure and public health outcomes.

Despite these limitations, the results provide consistent evidence of the spatial heterogeneity of dengue risk in Manta and constitute a solid basis for guiding the planning of interventions at the neighborhood level.

### 4.6. Future Research Directions

Although the model produces risk maps with well-defined suitability zones, it relies on passive surveillance data (i.e., routinely reported dengue cases) and static covariates. The incorporation of time-varying entomological indices, human mobility patterns and climate-change scenarios would facilitate dynamic forecasting and long-term planning. Moreover, intervention trials that overlay our hotspot map on real-time vector-control activities could quantify the added value of spatial targeting for case reduction.

In addition, MaxEnt, as a presence-only habitat suitability model, estimates relative habitat favorability but does not generate residuals or error terms that can be directly analyzed for spatial correlation. This characteristic limits the ability to validate suitability predictions by examining residual spatial dependence.

Future research could improve model validation and inference by incorporating presence–absence or abundance data, which would allow the application of spatial regression models such as generalized additive models with spatial smoothers, spatial generalized linear mixed models, or Bayesian hierarchical spatial models. These models explicitly account for spatial correlation in residuals and can better capture complex spatial dependencies.

## 5. Conclusions

The findings in this study show that high-resolution spatial modeling for identifying dengue transmission hotspots may be used for precision-targeted vector control strategies in Manta city, which shows that dengue is highly spatially heterogeneous. In addition, the coastal buffering effect, likely driven by microclimatic factors such as reduced rainfall and sea-breeze desiccation, indicates the complex interplay between environmental and infrastructural determinants for dengue transmission in coastal areas.

The model shows that population density is the primary driver of dengue cases suitability, reinforcing the epidemiological importance of urban planning interventions addressing water storage practices, waste management, and housing quality.

Dengue control strategies in similar coastal areas should integrate dynamic data sources, including entomological surveillance, mobility trends, and climate to forecasts and support adaptive public health planning.

## Figures and Tables

**Table 1:** Explanatory variables used in the MaxEnt habitat suitability model for dengue transmission in Manta, Ecuador, which include both environmental and socioeconomic indicators.

Explicative Variable	Measure Unit	Time	Time Unit
Elevation	m	2000	Year
Precipitation	mm	2018–2021	Monthly
Vegetation index	[−1.0 to +1.0]	2018–2021	16 days
Day temperature	°C	2018–2021	Monthly
Night temperature	°C	2018–2021	Monthly
Population	Density (inhab./ha)	2020 (projection)	Census
Sewer system access	% without sewer access	2010	Census
Drinking water access	% without water access	2010	Census
Housing condition	% of population in poorly maintained housing	2010	Census
Rivers	Distance to rivers	2020	Year

**Table 2:** Summary metrics by high, medium, and low suitability zones for dengue transmission in Manta city, in particular area, area proportion, population affected, number of reported dengue cases, and case density (cases per hectare).

Favorable Zones	Area ha (%)	Population Affected (%)	Cases (%) *	Case Density (Cases/ha)
High	957.38 (17.87)	118,409 (47.21)	84 (58.33)	0.088
Medium	1191.36 (22.24)	96,752 (38.58)	42 (29.17)	0.036
Low	3209.27 (59.90)	35,635 (14.21)	18 (12.5)	0.006
Total	5358 (100)	250,796 (100)	144 (100)	—

* Four georeferenced cases were excluded from this analysis because they were outside the defined neighborhood polygons.

**Table 3:** Percentage contribution of explanatory variables in the MaxEnt habitat suitability model for dengue transmission.

Variable	Percent Contribution (%)
Population	72.3
Sewer system access	13
Distance to rivers	2.5
Night temperature	2.4
Drinking water access	2.4
Precipitation	1.9
Housing condition	1.9
Vegetation index	1.6
Elevation	1.2
Day temperature	0.7

## Data Availability

The data used for modeling and analysis in this study are publicly available from the following sources: Elevation: Shuttle Radar Topography Mission (SRTM), available at USGS EROS Center [18]. Precipitation: Integrated Multi-satellite Retrievals for GPM (IMERG), available at NASA GPM [19]. Vegetation Index: MOD13Q1 Product (MODIS), available at USGS MODIS [20]. Day and Night Temperature: MOD11C3 Product (MODIS), available at USGS MODIS [21]. Population Projections: Instituto Nacional de Estadística y Censos (INEC), available at INEC Ecuador [22]. Access to Sewer and Drinking Water Services and Housing Condition: 2010 National Census, INEC [23]. Rivers: Land Cover and Agricultural Production Systems Map, Open Data Portal of the Government of Ecuador [24].

## Supplementary Materials

The following supporting information can be downloaded at: https://www.mdpi.com/article/10.3390/ijerph22101521/s1, File S1: The input values of all variables summarized by neighborhood.

## Abbreviations

The following abbreviations are used in this manuscript:

MSP	Ministry of Public Health
INEC	Instituto Nacional de Estadísticas y Censos
PCA	Principal Component Analysis
MaxEnt	Maximum Entropy Model
GIS	Geographic Information System
ROC	Receiver Operating Characteristic
SDM	Spatial Decision Modeling
AUC	Area Under the Curve

## References

[1] World Health Organization (WHO) Dengue and Severe Dengue. https://www.who.int/news-room/fact-sheets/detail/dengue-and-severe-dengue.

[2] Katzelnick L.C., Quentin E., Colston S., Ha T.-A., Andrade P., Eisenberg J.N.S., Ponce P., Coloma J., Cevallos V. (2024). Increasing Transmission of Dengue Virus across Ecologically Diverse Regions of Ecuador and Associated Risk Factors. PLoS Negl. Trop. Dis..

[3] Ministerio de Salud Pública (MSP) Gacetas Vectoriales. https://www.salud.gob.ec/gacetas-vectoriales/.

[4] Talbot B., Sander B., Cevallos V., González C., Benítez D., Carissimo C., Carrasquilla Ferro M.C., Gauto N., Litwiñiuk S., López K. (2021). Determinants of Aedes Mosquito Density as an Indicator of Arbovirus Transmission Risk in Three Sites Affected by Co-Circulation of Globally Spreading Arboviruses in Colombia, Ecuador and Argentina. Parasit. Vectors.

[5] Instituto Nacional de Estadística y Censos (INEC) Censo Ecuador 2022. https://geo.cepal.org/censo-ecuador/.

[6] Phillips S.J., Anderson R.P., Schapire R.E. (2006). Maximum Entropy Modeling of Species Geographic Distributions. Ecol. Model..

[7] Wisz M.S., Hijmans R.J., Li J., Peterson A.T., Graham C.H., Guisan A., NCEAS Predicting Species Distributions Working Group (2008). Effects of Sample Size on the Performance of Species Distribution Models. Divers. Distrib..

[8] Júnior P.D.M., Nóbrega C.C. (2018). Evaluating collinearity effects on species distribution models: An approach based on virtual species simulation. PLoS ONE.

[9] Richman R., Diallo D., Diallo M., Sall A.A., Faye O., Diagne C.T., Dia I., Weaver S.C., Hanley K.A., Buenemann M. (2018). Ecological Niche Modeling of Aedes Mosquito Vectors of Chikungunya Virus in Southeastern Senegal. Parasit. Vectors.

[10] Eastman J.R. TerrSet Geospatial Monitoring and Modeling System. https://www.clarku.edu/centers/geospatial-analytics/terrset/download/.

[11] Elith J., Phillips S.J., Hastie T., Dudík M., Chee Y.E., Yates C.J. (2011). A Statistical Explanation of MaxEnt for Ecologists: Statistical Explanation of MaxEnt. Divers. Distrib..

[12] Sánchez-Hernández D., Aguirre-Salado C.A., Sánchez-Díaz G., Aguirre-Salado A.I., Soubervielle-Montalvo C., Reyes-Cárdenas O., Reyes-Hernández H., Santana-Juárez M.V. (2021). Modeling Spatial Pattern of Dengue in North Central Mexico Using Survey Data and Logistic Regression. Int. J. Environ. Health Res..

[13] Gama Z.P., Yanuwiadi B., Rahayu P., Khalil R.J., Assiddiqy M.F., Rijalullah M.A., Kurniawan N. (2025). Present and Future Distribution Model Using MaxEnt: A Risk Map for Dengue Haemorrhagic Fever Based on Aedes Aegypti Mosquitoes Distribution in Malang Region, East Java, Indonesia. J. Trop. Biodivers. Biotechnol..

[14] Ordoñez-Sierra R., Mastachi-Loza C.A., Díaz-Delgado C., Cuervo-Robayo A.P., Fonseca Ortiz C.R., Gómez-Albores M.A., Medina Torres I. (2020). Spatial Risk Distribution of Dengue Based on the Ecological Niche Model of Aedes Aegypti (Diptera: Culicidae) in the Central Mexican Highlands. J. Med. Entomol..

[15] Ramasamy R., Surendran S.N. (2012). Global Climate Change and Its Potential Impact on Disease Transmission by Salinity-Tolerant Mosquito Vectors in Coastal Zones. Front. Physiol..

[16] Pan American Health Organization (PAHO) Handbook for Integrated Vector Management in the Americas. https://iris.paho.org/handle/10665.2/51759.

[17] Ministerio de Salud Pública (MSP) MSP desarrolló la Minga Nacional “Todos Contra el Dengue” Para Promover la Corresponsabilidad Colectiva. https://www.salud.gob.ec/msp-desarrollo-la-minga-nacional-todos-contra-el-dengue-para-promover-la-corresponsabilidad-colectiva/.

[18] United States Geological Survey (USGS) Shuttle Radar Topography Mission (SRTM). https://earthexplorer.usgs.gov/.

[19] NASA Goddard Earth Sciences Data and Information Services Center (GES DISC) GPM IMERG Global Precipitation Data. https://gpm1.gesdisc.eosdis.nasa.gov/data/GPM_L3/GPM_3IMERGM.07/.

[20] United States Geological Survey (USGS) MODIS Vegetation Index MOD13Q1. https://search.earthdata.nasa.gov/search?q=C1748066515-LPCLOUD.

[21] United States Geological Survey (USGS) MODIS Land Surface Temperature MOD11C3. https://search.earthdata.nasa.gov/search?q=C2565788897-LPCLOUD.

[22] Instituto Nacional de Estadística y Censos (INEC) Estimaciones y Proyecciones de Población. https://www.ecuadorencifras.gob.ec/proyecciones-poblacionales/.

[23] Instituto Nacional de Estadística y Población y Demografía Censo de Población y Vivienda 2010. https://www.ecuadorencifras.gob.ec/censo-de-poblacion-y-vivienda/.

[24] Ministerio de Agricultura y Ganadería, Ecuador Mapa de Cobertura y uso de la Tierra y Sistemas Productivos Agropecuarios Versión Editada 2020. https://datosabiertos.gob.ec/dataset/mapa-de-cobertura-y-uso-de-la-tierra-y-sistemas-productivos-agropecuarios.

